# What Does the Public Know About Varying Depression Severity?–Results of a Population Survey

**DOI:** 10.3389/ijph.2021.607794

**Published:** 2021-05-13

**Authors:** Anna Christin Makowski, Martin Härter, Georg Schomerus, Olaf von dem Knesebeck

**Affiliations:** ^1^ Institute of Medical Sociology, University Medical Center Hamburg-Eppendorf, Hamburg, Germany; ^2^ Department of Medical Psychology, University Medical Center Hamburg-Eppendorf, Hamburg, Germany; ^3^ Department of Psychiatry and Psychotherapy, University Hospital Leipzig, Leipzig, Germany

**Keywords:** depression literacy, severity, diagnosis, treatment options, general population, Germany

## Abstract

**Objectives:** In this study, we examine the public’s knowledge about different levels of depression severity in Germany.

**Methods:** Data stem from a national telephone survey in Germany. A total of 1,009 persons participated, response rate was 46.8%. A vignette was presented with signs of mild, moderate or severe depression. Participants were asked what they think the person has, which persons and services are helpful and how effective different treatment options are. Differences between the three vignettes were tested with 95% confidence intervals and *χ* ^2^-tests.

**Results:** 55.3% of the respondents identified depression as the health problem in question. Participants who heard the vignette with moderate symptomatology recognized depression more often. Across groups, a general practitioner was named most frequently concerning helpful persons/services. Effectiveness of psychotherapy received high levels of approval, online therapy and books were less often rated as effective. There were only few significant differences between the three vignettes.

**Conclusions:** This is the first study examining public depression literacy for different severity levels. Small differences between severity levels indicate a lack of knowledge, which may have adverse consequences for adherence to treatment, especially for mild depression.

## Introduction

The concept of mental health literacy (MHL) was first introduced by Jorm and colleagues and relates to public knowledge and beliefs about the recognition, management, and prevention of mental disorders [1]. This work has been constantly developed and extended by further components. Accordingly, MHL also includes the ability to notice a developing mental illness, the knowledge of help or treatment options, as well as the ability to self-help or support others affected by a mental health problem [2]. The conceptual model that laypersons use to understand and explain mental disorders influences how they deal with psychiatric symptoms when they themselves or others in their environment are affected. It has been shown that difficulties in identifying a mental illness and lack of knowledge about mental health services constitute barriers to seeking help [3]. In contrast, greater MHL predicts willingness to disclose one’s mental health problems and take up treatment [4, 5], and is positively associated with adherence to treatment [2]. The ability to recognize a mental health problem also affects preferences in treatment options, i.e., the choice of professional help-seeking over lay-help seeking [6, 7].

By now, there is an extensive body of research in the field of MHL, and it has been investigated with regard to different mental disorders [8]. Concerning depression literacy, international literature has shown an increase in the correct identification of depression over time. Recent population studies using vignettes elicited recognition rates between 55 and 79% [7, 9–11]. When asked for their assessment of treatability or prognosis of disease, respondents stated that they think depressive disorders can be treated relatively well and that they generally have a good prognosis [9, 12, 13].

A systematic literature review indicated that professional help for mental health problems has a relatively high reputation among the general population [14]. With reference to depression [14], it was found that the public tends to favor mental health professionals when having to rank sources of help, but strongly agrees to a general practitioner (GP) when asked in an open-ended question.

Regarding the effectiveness of different treatment options for depression, about 93% of respondents stated that they thought psychotherapy is ‘rather/very effective’. This held true for a share of around 92% concerning own activities (physical exercise, relaxation methods). Medication was thought to be effective by 76% of the respondents [13]. In a study by [12]; among others, a counselor, drugs prescribed by a psychiatrist, and relaxation were met with high levels of agreement.

Overall, the current state of research shows that the general public displays relatively good depression literacy. However, the research results refer to depression in general, without taking the severity of depressive syndromes into account.

To our knowledge, there are no studies on public knowledge of depressive syndromes of varying severity. In this study, we aim to examine the German public’s depression literacy on three different levels of depression severity: minor, moderate, and severe depression.

## Methods

### Study Design and Sample

Data stem from a nationwide telephone survey (computer-assisted telephone interview, CATI) conducted between November 2019 and January 2020 in Germany. The random sampling procedure was based on data provided by the Association of German Market and Social Research (ADM). This is published key data for the number range that the Federal Network Agency makes available to telephone providers. In principle, this number range comprises all telephone numbers that can be used in the Federal Republic of Germany, also including non-registered telephone numbers via random digital dialing [15]. From this number range, a random sample was drawn. To include mobile-only users and target groups rather difficult to reach, the study design followed a dual-frame approach, i.e., 30% of the gross sample consisted of mobile numbers. To ensure a sample representative of the German population, all regions in Germany were included.

Trained interviewers called on different days of the week, repeated calls were made on eight occasions before a number dropped out. In the case of an established connection via landline, the target person was randomly selected via Kish-selection grid [16]. In this method, all persons of the target group (at least 18 years of age) were first identified by the contact person who answered the telephone. Then the Kish-selection grid randomly selected the person to be interviewed. For mobile users, the target person was automatically the owner or main user of the phone. If this person was younger than 18 years, it was considered a neutral drop-out.

In this study, different case stories (vignettes) are used. A sample size calculation showed that a number of *n* = 330 participants per vignette (i.e., *N* = 1,000) is sufficient to identify small sized differences with a statistical power of 80% and a Type-I error of 0.05 when comparing vignettes with regard to categorical outcomes using *χ* ^2^-tests. Additionally, previous studies with a similar design have shown this number of respondents to be adequate to detect significant differences in public attitudes [17, 18]. The net sample of the study consisted of *N* = 2,154 randomly selected persons. Of these, *N* = 625 (29.0%) could not be reached, and *n* = 520 (24.1%) refused to participate. This led to an overall sample of *N* = 1,009 participants, with a corresponding response rate of 46.8%.

The Local Psychological Ethics Committee at the Center for Psychosocial Medicine, University Medical Center Hamburg approved this study (No. LPEK-0091). Since the interviews were telephone-based, the respondents were verbally informed about the study and asked for consent to participate. Participants’ consent and refusal were documented.

### Vignettes

In the present study, three unlabeled case-vignettes were used, each presenting signs and symptoms indicative of different degrees of depression (mild, moderate, and severe, see “Appendix”). This resulted in the following numbers of participants: mild depression *n* = 353, moderate depression *n* = 334, and severe depression *n* = 322.

The case stories were developed in cooperation with psychiatrists and clinical psychologists on the basis of the International Classification of Diseases, 10th edition [19] and the National Clinical Care Guideline for Depression [20]. In these documents, different degrees of depression are specified depending on the number and severity of symptoms presented. An example for a moderate depressive episode would be a patient suffering from two main symptoms for depression (e.g. lowered mood, loss of interest) in combination with three to four additional symptoms (e.g. lack of concentration, reduced self-esteem, disturbed sleep) for at least two weeks. A mild depressive episode is characterized by two main and two additional symptoms, while a severe depressive episode is defined by three main symptoms and four or more additional symptoms for at least two weeks. The vignettes were audio-recorded by a trained speaker. To neutralize interviewer-associated effects, the audio files were directly played to the respondents from the computer. Gender of the fictive patient in the vignette was systematically varied.

### Measures

In terms of knowledge and beliefs, different indicators were used. After presentation of the vignette, respondents were asked what they think the person in the case study has. This approach has also been applied in previous research on knowledge about and attitudes toward mental disorders (see e.g., [21]). Answers to this open-ended question were discussed and coded by two researchers. For the analyses, we counted the respondents who recognized a depression and the different grades of severity. Additionally, the participants were asked whether they thought the disorder is treatable (ranging from 1 ‘not at all’ to 4 ‘very well’) [22]. For further analyses, categories were dichotomized, to identify those respondents who consider the disorder well or very well treatable.

In the further course of the interview, respondents were asked who or where they thought the person should turn to for help and name up to three persons or services to go to they deemed most helpful. Again, the answers to this open-ended question were discussed and coded. Some answers stood on their own, e.g. GP, psychologist, family, and friends. Others, such as different specific counseling services were grouped in a generic category ‘counseling service’. In this study, we present those categories which were stated by at least 10% of the respondents.

Moreover, we asked the respondents to rate the effectiveness of eight different treatment options on a scale from 1 ‘not effective at all’ to 4 ‘very effective’ (psychotherapy, physical activity, participation in self-help groups, treatment in a specialized clinic, relaxation methods, medication, reading books and brochures, and services on the internet such as online therapy). The chosen treatments are based on recommendations of the National Clinical Care Guideline Depression for different grades of severity in depression [20]. For further analyses, categories were dichotomized by combining the first two (not at all effective/rather not effective) and the last two response options (rather/very effective).

### Statistical Analyses

Analyses were conducted using the statistical program packages SPSS [23] and R [24]. To test for differences between groups regarding sociodemographic variables, *χ* ^2^-tests were computed in SPSS. For the difference tests regarding the three vignettes, 95% confidence intervals (95 CI) as well as *χ* ^2^-tests were calculated in R. To test for the overall difference between severity levels, all three groups were tested in a 3 by 2 table. Then 2 by 2 tables were applied in order to test for differences between each group of severity level. To encounter multiple testing, all p-values were adjusted according to [25]; exact p-values are reported.

## Results

The sociodemographic characteristics of the sample are briefly described in Table 1. In terms of gender, age, and level of education, the distribution is similar to that in the general German population as reported by official statistics [26, 27].

**TABLE 1:** Sociodemographic variables of the sample compared to official statistics (Germany 2020).

	Sample (*N* = 1,009)	Official statistics	*p* (*χ* ^2^)
Gender			
Female	51.1	50.7^a^	0.486
Age groups			
18–≤24 years	6.8	9.1^b^	0.518
25–≤39 years	13.4	21.2^b^	
40–≤59 years	35.8	33.8^b^	
60–≤64 years	12.6	10.1^b^	
≥ 65 years	31.4	25.8^b^	
Level of education			
≤ 9 years	35.9	35.7^c^	0.050
10 years	30.6	30.9^c^	
≥ 12 years	33.5	33.1^c^	

a Federal Office of Statistics (Destatis) 2019 p 26.

b Federal Office of Statistics (Destatis) 2019 p 31.

c Federal Office of Statistics (Destatis) 2018 p 21; weighted data.

The majority of the sample thought that the person is suffering from some form of depression in general. Only few respondents stated that this was a mild (7.5%), moderate (0.1%), or severe (0.9%) depression. Thus, when speaking of recognition of depression in the following, we mean those respondents who identified some form of depression in general plus those cases who stated a grade of severity of depression. This leads to a share of 55.3% of participants (Table 2). The difference between participants who heard a mild depression vignette compared to those with a moderate depression vignette attained statistical significance (*p* = 0.0030). This also held true for the difference between mild and severe depression (*p* = 0.0052). Among those participants, who did not explicitly state depression as a possible diagnosis, most frequent answers were 'some form of mental illness', followed by 'burnout' and 'exhaustion/overload'. There was only one participant who answered 'don´t know'.

**TABLE 2:** Recognition of depression and beliefs about treatability in % with corresponding 95% CI (total sample and subgroups) (Germany 2020).

	Total sample	Mild depression	Moderate depression	Severe depression
Recognition of depression	*N* = 1,008	*n* = 352	*n* = 334	*n* = 322
	55.3 [52.2–52.4]	45.9 [40.7–51.1]^*^ ^1^	62.9 [57.7–68.1]	57.8 [52.4–63.2]^*^ ^2^
Disorder is treatable (well/very well)	*N* = 961	*n* = 329	*n* = 321	*n* = 311
	81.4 [78.9–83.9]	83.0 [78.9–87.1]	83.8 [79.8–87.8]	76.8 [72.1–81.5]

^*1^Significantly different from moderate depression (*χ* ^2^: p = 0.0030);

^*2^Significantly different from mild depression (*χ* ^2^: p = 0.0052).

Those presented with a severe course of depression tended to agree to a lesser extent that the disorder is well or very well treatable compared to participants who received a mild or a moderate depression vignette. However, this difference did not attain statistical significance (Table 2).

The number of persons or helpful services that were stated are presented in Table 3. Only 1.6% (17 participants) did not name any at all. The vast majority stated three helpful contacts the person in the vignette could turn to. There were statistically significant differences between the mild and severe vignettes regarding the number of respondents who did not name any helpful persons or services. When presented with a mild vignette, respondents tended to state less options.

**TABLE 3:** Number of helpful persons or services to go to in % with corresponding 95% CI (total sample and subgroups; open-ended question, participants were asked to name up to three options) (Germany 2020).

	Total sample (*N* = 1,009)	Mild depression (*n* = 353)	Moderate depression (*n* = 334)	Severe depression (*n* = 322)
Number of persons or contact points given				
0 (≙ “don’t know”)	1.6 [0.8–2.4]	3.4 [1.5–5.3]^*1^	0.6 [−0.2–1.4]^*2^	0.6 [−0.2–1.4]
1	4.4 [3.1–5.7]	5.1 [2.8–7.4]	4.5 [2.3–6.7]	3.4 [1.4–5.4]
2	10.2 [8.3–12.1]	11.4 [8.1–14.7]	9.3 [6.2–12.4]	9.9 [6.6–13.2]
3	83.8 [81.4–86.0]	80.1 [75.9–84.3]	85.6 [81.8–89.4]	86.1 [82.3–89.9]

^*1^Significantly different from severe depression (Fisher’s p = 0.0375);

* ^2^Significantly different from mild depression (Fisher’s p = 0.0375).

Table 4 lists eight categories of possible helpful persons or services to go to in descending order of frequency (when looking at the total sample). Irrespective of severity, the GP was stated most often. For subsequent categories, there were between-group differences in the ranking of services to call on. For example, in case of severe depression, family was stated more often as one of the three most helpful options than in mild or moderate depression. However, none of the differences between groups were statistically significant.

**TABLE 4:** Persons or places to go to deemed most helpful in % with corresponding 95% CI (total sample and subgroups; open ended question, participants were asked to name up to three options) (Germany 2020).

	Total sample (*N* = 993)	Mild depression (*n* = 340)	Moderate depression (*n* = 332)	Severe depression (*n* = 321)
General practitioner	56.8 [53.7–59.9]	60.6 [55.4–65.8]	53.9 [48.5–59.3]	55.8 [50.4–61.2]
Psychologist	38.8 [35.8–41.8]	35.3 [30.2–40.4]	42.6 [37.3–47.9]	38.6 [33.3–43.9]
Friends	35.1 [32.1–38.1]	36.2 [31.1–41.3]	34.9 [29.8–40.0]	34.3 [29.1–39.5]
Family	33.9 [31.0–36.9]	31.2 [26.3–36.1]	32.2 [27.2–37.2]	38.4 [33.1–43.7]
Counseling service	29.0 [26.2–31.8]	27.9 [23.1–32.7]	28.3 [23.5–33.2]	30.8 [25.8–35.9]
Psychiatrist	18.8 [16.4–21.2]	19.4 [15.2–23.6]	17.2 [13.1–21.3]	19.9 [15.5–24.3]
Medical specialist (except psychiatrist)	12.9 [10.8–15.0]	14.1 [10.4–17.8]	10.2 [6.9–13.5]	14.4 [10.6–18.2]
Psychotherapist	12.2 [10.2–14.2]	8.6 [5.7–11.7]	14.2 [10.4–17.8]	14.0 [10.2–17.8]

Respondents were also asked to rate the effectiveness of eight different treatment options. Results are presented in descending order (when looking at the total sample) in Table 5. Irrespective of severity, psychotherapy, physical activity, participation in self-help groups, treatment in a specialized clinic, and relaxation were considered effective by more than or around 80% of the respondents. More respondents tended to rate medication as effective when the syndrome gets more severe. However, the differences between groups were not statistically significant.

**TABLE 5:** Beliefs about effectiveness of treatment options in % with corresponding 95% CI (total sample and subgroups) (Germany 2020).

	Total sample (*N* = 982–1,007)	Mild depression (*n* = 339–353)	Moderate depression (*n* = 320–333)	Severe depression (*n* = 309–322)
Treatment options (rather/very effective)				
Psychotherapy	93.2 [91.6–94.8]	90.5 [87.4–93.6]	94.9 [92.5–97.3]	94.4 [91.9–96.9]
Physical activity	93.1 [91.5–94.7]	95.5 [93.3–97.7]	90.7 [87.6–93.8]	93.1 [90.3–95.9]
Self-help groups	89.0 [87.1–91.0]	84.6 [80.8–88.4]^*1^	89.7 [86.4–93.0]	93.1 [90.3–95.9]
Relaxation	83.2 [80.9–85.5]	83.4 [79.5–87.3]	86.5 [82.8–90.2]	79.4 [75.0–83.8]
Treatment in a specialized clinic	81.4 [79.0–83.8]	80.2 [76.0–84.4]	79.9 [75.6–84.2]	84.2 [80.2–88.2]
Medication	67.4 [64.5–70.3]	65.8 [60.8–70.9]	66.4 [61.3–71.5]	70.4 [65.4–75.5]
Books and brochures	38.8 [35.8–41.8]	41.1 [35.9–46.3]	34.5 [29.4–39.6]	40.9 [35.5–46.3]
Online therapy	18.2 [15.8–20.6]	24.0 [19.5–28.5]^*2^	15.9 [11.9–19.9]^*3^	14.2 [10.3–18.1]

^*1^Significantly different from severe depression (*χ* ^2^: p = 0.0003);

^*2^Significantly different from severe depression (*χ* ^2^: p = 0.0022);

* ^3^Significantly different from mild depression (*χ* ^2^: p = 0.0248).

Regarding mild depression, self-help groups were rated as less effective (*p* = 0.0003) when compared to severe depression. Services on the internet such as online therapy programs were rarely rated as rather or very effective, and these ratings decline by intensifying degree of depression. These differences also attained statistical significance when comparing mild to moderate (*p* = 0.0248) and mild to severe (*p* = 0.0022) depression.

## Discussion

To the best of our knowledge, this is the first study examining depression literacy with regard to mild, moderate, and severe symptomatology of disease in the general population. Using three different vignettes, we elicited public beliefs and knowledge regarding recognition of disorder as well as helpful persons and services and the assessment of effective treatment options.

The majority of respondents was able to recognize depression when listening to the reported syndromes, though without stating a grade of severity. When presented with a vignette describing a mild depression, respondents identified the disease significantly less often. No statistically significant differences were found regarding the open-ended question about helpful persons or services. The evaluation of efficient therapeutic measures revealed a relatively similar picture between groups, with only few significant differences depending on degree of severity.

There are quite a few studies on depression literacy that report the public’s ability to recognize depression [9, 28]. The share of respondents in our study that identified a depressive disorder in general can be compared with these findings. The correct disease recognition is a first, very important step toward help-seeking or providing support for other affected persons. From this point of view, the high proportion of identifications can be considered positive. However, we were also able to show that a mild depression was less frequently recognized as compared to moderate or severe depression. On the one hand, one could say this is acceptable, as mild courses of disease bear the lowest need indication for (immediate) treatment. On the other hand, early detection would be all the more important for timely professional help and to avoid a potentially unfavorable disease course [29].

The results on helpful persons or services in the case of depression did not show any significant differences between the different case severities. The GP plays a central role in all three degrees of depression. He or she was most often named by the respondents as a helpful person to go to. Here, patients with mild or moderate depression can receive evidence-based help. However, current research results also show that there is still a relevant shortfall in the care of patients with depressive disorders in the primary sector. For example, about 60% of all patients with depression were not treated with antidepressants or psychotherapy according to the guidelines in general practices [30]. Public opinion that a general practice is an important place to go was also evident in other studies. Research from Australia showed that 90% of participants rated the GP as helpful, followed by a counselor [31]. In our study, the GP was followed in second and third place by the psychologist or friends or family, depending on the degree of severity. The frequent mention of confidants from family and friends in mental illness has also been shown in other studies [9, 12]. It is obvious that, in the event of illness, people first turn to friends or family in the hope of receiving help and support. This means, however, that a social network has to exist, and that this network must have sufficient knowledge to be able to provide adequate help and advice for those affected.

We would have expected more differences in the assessment of effective treatment options. All in all, only few statistically significant differences emerged between groups, or showed a trend depending on severity. There are some measures, e.g. psychotherapy, which are considered helpful by almost all respondents, displaying a ceiling effect. With increasing severity, respondents were more inclined to assess self-help groups as effective. Depending on the individual case, it can be regarded beneficial to involve self-help groups in therapy [20]. However, it should not be seen as a stand-alone measure and is not always helpful, especially in severe cases. Although the benefits of self-help are indisputable, the effectiveness of self-help groups has not yet been sufficiently proven [32].

A similar gradient can be seen for medication, though non-significant. Compared to other treatment options, medication is not seen as effective. This reluctance has also been shown in other studies. Over the past years, a rather skeptical view on medication such as antidepressants has prevailed in the public [33, 34]. Our results indicate that this also holds true for severe forms of depression where medication is one of the most effective measures. Therapeutic measures such as bibliotherapy or online interventions were considered least effective, and there is a significant reverse gradient for online help. However, these are evidence-based treatments recommended by guidelines [20, 35] and frequently used in stepped care for mild or when in transition to moderate depression. So far, these options do not seem to be accepted as effective measures in the public.

This is the first study examining public depression literacy for different severity levels. For a number of years, the field of mental health literacy has focused on public knowledge related to individual diseases. Accordingly, some information is already available nationally and internationally, for example on the correct recognition of depression, the classification of treatability or the assessment of the effectiveness of various measures or helpful persons to turn to (e.g., [7, 9, 13, 14]). However, not all of these studies made use of vignettes to elicit public beliefs. When vignettes were employed, usually only one case story was used, which then tended to depict the clinical picture of moderate depression. Consequently, little is known to date about public knowledge of a mild or severe course of depression. Since early detection and initiation of treatment are also of great importance in mild depression, the present study is an innovative approach that can make a clear contribution to the state of current research in this area. Overall, our results indicate that beliefs about disease and treatment hardly differ between varying severity levels of depression.

### Limitations

When evaluating our findings, some methodological aspects have to be mentioned and discussed. Our analyses are based on a carefully drawn random sample and a response rate of 46.8% is in line with participation rates of other studies [36]. Nevertheless, we cannot rule out selection bias due to non-response. Non-responders may have different beliefs regarding severity of depression and possible treatment options. However, the comparison of social demographic characteristics in the sample with official statistics indicates that selection bias is limited in this regard. A design using vignettes to elicit beliefs among the general public is frequently applied in population-based attitude research in mental health. The vignettes should not be too long to be included in surveys. In turn, this can mean that the case stories may be too short to convey a holistic picture of the individual with depression, or that they could not be kept in mind throughout the whole interview. Experts in the field as well as recommendations from national guidelines were consulted when developing the case stories of varying depression severity. This ensures that the levels of severity are displayed correctly. However, when asked for what they think the person in the vignette has, hardly any participant named a degree, but rather stated depression in general. Whether this means that different degrees of severity are not known to the general public, or are known, but were not thought to be of importance in this context, cannot be said. One might argue that this is an unfavorable circumstance when setting out to evaluate differences that are based on different degrees of severity. However, the vignettes in our study were non-labeled, i.e., participants were not informed about the ‘correct’ diagnosis of the person in the vignette. We assumed that respondents would come to different conclusions when presented with varying symptomatology.

## Conclusion

Previous research on depression literacy has referred to depression in general, without taking into account different severity levels. As mental health literacy predicts disclosure of illness and help-seeking [2, 4], the public knowledge on depression and its different manifestations is of great relevance. According to national and international guidelines, most treatment recommendations are based on depression severity [20, 35]. Accordingly, treatment options of varying intensity are applied depending on the patient’s diagnosis and tailored to her or his specific needs. Our results indicate that there is a lack of public knowledge and acceptance regarding such treatment approaches which may have unfavorable consequences for adherence. Some interventions that are recommended as evidence-based in the professional community (e.g., online therapy) obviously are not rated as effective by large parts of the general population. In this regard, depression literacy may be improved by psychoeducational interventions. From 2011 to 2014, there was a public awareness campaign targeting information and awareness of depression and other mental health problems in Hamburg, Germany [37]. An evaluation of mental health literacy in the framework of this project showed that people who had been aware of the campaign displayed improved knowledge [38]. This underlines the possible effectiveness of such psychoeducational approaches. However, the content of the public campaign referred to depression in general, and no gradation into severity levels was made.

## Data Availability

The datasets presented in this article are not readily available because we would like to make the dataset available upon reasonable request. Requests to access the datasets should be directed to AM, a.makowski@uke.de

## Appendix

### Minor Depression

37-year-old Denise D.* has been feeling down on and off for the past few months. Mrs. D. has little interest in everyday things.

She sometimes finds it difficult to fall asleep, and at work Mrs. D. can no longer concentrate very well. In everyday life Mrs. D. is impaired, but she can cope with most activities.

### Moderate Depression

37-year-old Denise D.* has often felt so down for the past few months that nothing can cheer her up. Mrs. D. has lost interest in everyday things.

She often has trouble falling asleep in the evenings and often feels tired and weak in the mornings. At work Mrs. D. has difficulty concentrating. Mrs. D. feels that she is not good enough. Overall, Mrs. D. has great difficulty coping with her everyday life.

### Severe Depression

For several months now, 37-year-old Denise D.* has been feeling so down all the time that nothing can cheer her up. Mrs. D. no longer has anything that she enjoys or is happy about.

It takes her a long time to fall asleep in the evenings and she wakes up frequently at night. In the mornings she is always tired and powerless. If she manages to go to work, she can no longer concentrate at all. Mrs. D. has the feeling that she is not good enough and she doubts that her life still makes any sense at all.
